# Reduced Risk of Reoperations With Modern Deep Brain Stimulator Systems: Big Data Analysis From a United States Claims Database

**DOI:** 10.3389/fneur.2021.785280

**Published:** 2021-12-02

**Authors:** Chengyuan Wu, Sean J. Nagel, Rahul Agarwal, Monika Pötter-Nerger, Wolfgang Hamel, Ashwini D. Sharan, Allison T. Connolly, Binith Cheeran, Paul S. Larson

**Affiliations:** ^1^Department of Neurological Surgery, Vickie and Jack Farber Institute for Neuroscience, Thomas Jefferson University, Philadelphia, PA, United States; ^2^Department of Neurological Surgery, Center for Neuro-Restoration, Cleveland Clinic, Cleveland, OH, United States; ^3^Abbott Laboratories, Austin, TX, United States; ^4^Department of Neurology, University Medical Center Hamburg-Eppendorf, Hamburg, Germany; ^5^Department of Neurological Surgery, University Medical Center Hamburg-Eppendorf, Hamburg, Germany; ^6^Department of Neurological Surgery, Weill Institute for Neurosciences, University of California, San Francisco, San Francisco, CA, United States

**Keywords:** deep brain stimulation, Parkinson's disease, essential tremor, Medicare, big data & analytics, neurosurgery, complications, surgical risk

## Abstract

**Objective:** There have been significant improvements in the design and manufacturing of deep brain stimulation (DBS) systems, but no study has considered the impact of modern systems on complications. We sought to compare the relative occurrence of reoperations after *de novo* implantation of modern and traditional DBS systems in patients with Parkinson's disease (PD) or essential tremor (ET) in the United States.

**Design:** Retrospective, contemporaneous cohort study.

**Setting:** Multicenter data from the United States Centers for Medicare and Medicaid Services administrative claims database between 2016 and 2018.

**Participants:** This population-based sample consisted of 5,998 patients implanted with a DBS system, of which 3,869 patients had a *de novo* implant and primary diagnosis of PD or ET. Follow-up of 3 months was available for 3,810 patients, 12 months for 3,561 patients, and 24 months for 1,812 patients.

**Intervention:** Implantation of a modern directional (MD) or traditional omnidirectional (TO) DBS system.

**Primary and Secondary Outcome Measures:** We hypothesized that MD systems would impact complication rates. Reoperation rate was the primary outcome. Associated diagnoses, patient characteristics, and implanting center details served as covariates. Kaplan–Meier analysis was performed to compare rates of event-free survival and regression models were used to determine covariate influences.

**Results:** Patients implanted with modern systems were 36% less likely to require reoperation, largely due to differences in acute reoperations and intracranial lead reoperations. Risk reduction persisted while accounting for practice differences and implanting center experience. Risk reduction was more pronounced in patients with PD.

**Conclusions:** In the first multicenter analysis of device-related complications including modern DBS systems, we found that modern systems are associated with lower reoperation rates. This risk profile should be carefully considered during device selection for patients undergoing DBS for PD or ET. Prospective studies are needed to further investigate underlying causes.

## Strengths and Limitations of This Study

- Strict inclusion and exclusion criteria provide a reliable comparison between two different deep brain stimulator systems.
- Use of the Centers for Medicare and Medicaid Services (CMS) administrative claims database provides a large volume of data for analysis.
- The CMS database provides objective data from multiple centers within the United States.
- Analysis is limited to the information within the CMS database because more in-depth clinical data was not available to provide a more complete picture of each clinical scenario.
- The retrospective nature of the study prevents the ability to control all confounding variables.

## Introduction

Deep brain stimulation (DBS) is a highly effective treatment for movement disorders like essential tremor (ET) and Parkinson's disease (PD). While multiple randomized controlled trials have demonstrated the superiority of DBS over best medical therapy in PD, it is associated with approximately twice as many complications (1). This risk–benefit ratio plays a significant role in willingness to consider DBS implantation and may contribute to its current underutilization (2).

Although numerous studies have described DBS complications, reporting has been inconsistent—with rates ranging between 0 and 20% and with exact values not being reported at times (3). Furthermore, studies have been limited to traditional omnidirectional (TO) DBS systems. No study has considered the impact of modern DBS systems, which include a number of innovations. While much attention has been paid to segmented lead design allowing for stimulation field shaping (4, 5), modern systems also differ in design and construction of its other components. We, therefore, sought to compare the relative occurrence of complications necessitating reoperation after *de novo* implantation of modern directional (MD) and TO DBS systems in patients with PD and ET in the United States.

## Materials and Methods

We conducted an observational, non-randomized, retrospective, contemporaneous cohort study of Medicare patients undergoing DBS implantation in the United States. Data from the Centers for Medicare and Medicaid Services (CMS) administrative claims database were analyzed to identify DBS implantations performed in patients with PD and ET. Revision and removal procedures performed within 2 years of follow-up were compared between an MD and TO DBS systems.

### Data Sources

Eligibility, baseline characteristics, and outcomes were derived from CMS longitudinal administrative claims files between 2013 and 2019. Patients who underwent implantation between October 6, 2016 and December 31, 2018 were included. This was a unique period during which the Abbott Infinity™ system was the only commercially available MD system in the United States. Cross-referencing between the Abbott Patient Device Tracking (PDT) database and the CMS database allowed classification of the subgroups of interest. The use of CMS data and linkage to the PDT database were approved through a data use agreement (RSCH-2019-53524) with CMS. This agreement was established with Abbott, who provided funding for this study.

### Ethics Approval

The research protocol was approved by Western Institutional Review Board (IRB) with a waiver of informed consent and a HIPAA waiver.

### Patient and Public Involvement

As this was retrospective cohort study performed on anonymized data, it was not possible to involve patients in the design, conduct, or reporting of this study.

### Medicare Claims Files

The CMS database consists of administrative claims for healthcare encounters including inpatient hospital, outpatient hospital, and physician claims. Relevant International Classification of Diseases procedure (ICD-10-PCS) and diagnosis (ICD-10-CM) codes, and Current Procedural Terminology (CPT) codes were extracted to identify relevant diagnoses and procedures (Table 1).

**Table 1:** List of procedure codes defining reoperations and diagnosis codes used for classifying reason for complication.

***Lead Implant procedure codes***	
For inclusion	
ICD-10-PCS	00H00MZ, 00H03MZ, 00H04MZ
CPT	61863, 61864 (without MER) 61867, 61868 (with MER)
***IPG Implant procedure codes***	
For inclusion	
ICD-10-PCS	0JH60BZ, 0JH60DZ, 0JH60EZ, 0JH63BZ, 0JH63DZ, 0JH63EZ, 0JH70BZ, 0JH70DZ, 0JH70EZ, 0JH73BZ, 0JH73DZ, 0JH73EZ, 0JH80BZ, 0JH80DZ, 0JH80EZ, 0JH83BZ, 0JH83DZ, 0JH83EZ
CPT	61885, 61886
***Lead/IPG Revision/Removal procedure codes***	
For exclusion	
ICD-10-PCS	00P00MZ, 00P03MZ, 00P04MZ, 00P0XMZ 00W00MZ, 00W03MZ (lead) 0JPT0MZ, 0JPT3MZ, 0JWT0MZ, 0JWT3MZ (IPG)
CPT	61880 (lead), 61888 (IPG)
***DBS Analysis and Programming procedure codes***	
For exclusion	
CPT	95978, 95979, 95983, 95984, 95970
***Diagnosis codes (ICD-10-CM)***	
For inclusion	
Parkinson's disease	G20, G21.11, G21.19, G21.2, G21.3, G21.8, G21.9, G23.1, G31.83, G31.85
Essential tremor	G25.0, G25.1, G25.2
**Cause of complications (ICD-10-CM)**	
When present on primary diagnosis or other diagnosis code associated with one of the procedures listed above	
**Infection** *Includes infection of lead, IPG, or other, when associated with a revision or removal procedure*	T8140XA, T8140XD, T8141XA, T8141XS, T8142XA, T8142XD, T8143XA, T8149XA, T814XXA, T814XXD, T814XXS, T8460XA, T85731A, T85731D, T85734A, T85738A, T85738S, T8579XA, T8579XD, T8579XS, A414, A419, A4901, B451, B9562, B965, B999, G060, G08, L03313, L089, S0100XA, S0102XA, T8130XA, T8130XD, T8131XA, T8131XD, T8131XS, T8132XA, T8132XD, T85732A, T85733D
**Hardware malfunction** *Includes hardware break, displacement/migration, various mechanical complications, adjustment, and erosion*	T82110A, T85110A, T85110D, T85110S, T85111A, T85113A, T85113D, T85113S, T85118A, T85118D, T85118S, T85120A, T85120D, T85120S, T85121A, T85123A, T85128A, T85190A, T85190D, T85190S, T85193A, T85193D, T85193S, T85199A, T85199D, T85615A, T85618A, T85618D, T85625A, T85628A, T85695A, T85695D, T85695S, T85698A, T85698D, T85890A, T85890D, T85890S, T82897A, T85191A, T85898A, T8589XA, T8589XD, T859XXA, Z4542, Z4549, Z4589, Z459, Z462
**Other/Unidentified**	Any procedure not having one of the above diagnosis codes

ICD-10-PCS: International Classification of Diseases, Tenth Revision, Procedure Coding System; ICD-10-CM: International Classification of Diseases, Tenth Revision, Clinical Modification; CPT: Current Procedural Terminology; IPG: implantable pulse generator; MER: microelectrode recording.

The CMS Master Beneficiary Summary File (MBSF) provided unique patient characteristics of age, sex, geographical location, race or ethnicity, and date of death. Each patient was assigned a unique, anonymized identifier that allowed longitudinal tracking of healthcare encounters *via* CMS claims files on the Virtual Research Data Center (VRDC) (6).

### Patient Device Tracking Data

The PDT database contains the device models associated with the intracranial lead and the implantable pulse generator (IPG) components of the DBS system. It also contains patient characteristics and implant details such as implant date and implanting center. These secondary patient identifiers were used to create a unique, anonymized identifier for each study patient.

### Patient Selection

We identified patients with a Medicare claim containing a procedure code for a DBS lead implant between the aforementioned dates. To determine if a procedure was a *de novo* implant, CMS data for at least 12 months prior to the lead implantation were analyzed to ensure the absence of any DBS-related claims (e.g., prior DBS implant, revision/removal, or programming/analysis). Furthermore, the subsequent DBS IPG implantation had to occur within 3 months of the lead implantation. DBS lead implants fulfilling both criteria were considered an *index lead implant*, whereas those with insufficient data to confirm adherence to both criteria were excluded. This approach maximized the likelihood of including only *de novo* implantations. Only patients with a primary diagnosis of PD or ET for the index lead implant were included, as other DBS indications were not shared among TO and MD systems.

Longitudinal CMS data were analyzed with a minimum follow-up of 3 months (acute period) and a maximum follow-up of 2 years after index lead implant, censoring for death, end of Medicare enrollment, or end of data availability.

### Linking Methodology to Identify Modern Directional DBS Systems

The DBS IPG implants identified in the CMS database were linked with implants from the PDT database using previously described probabilistic linkage methods (7). Implanted patients whose study identification codes matched uniquely across CMS and PDT databases were allocated to the MD subgroup. Approximate matches were removed from further analysis to avoid modestly probable misclassification. All the remaining patients were allocated to the TO subgroup.

### Patient Characteristics and Covariates

The age of patient age at implant and sex were acquired from the MBSF. Indication for implant was determined from the primary diagnosis associated with the index lead implant claim.

Pertinent comorbidities were derived from conditions listed in the Chronic Conditions Warehouse (CCW) section of the MBSF and in the Elixhauser comorbidity system (8, 9). While blinded to their prevalence in the cohort, it was determined that atrial fibrillation, diabetes, heart failure, hypertension, stroke, obesity, and weight loss were the most clinically relevant in DBS surgery. Using claims data prior to the index lead implant, patient characteristics were recorded as frequencies and percentages for categorical variables and as means with SD for continuous variables. Statistical differences in the patient characteristics were tested using the *t*-test for continuous variables and the chi-square test for categorical variables.

Implanted DBS systems were classified as *unilateral* or *bilateral* based on the number of leads implanted on the day of the index lead implant and within the acute period. CPT modifiers for *bilateral, multiple*, or *distinct* leads were also used to facilitate classification. Unilateral systems included patients implanted with a single lead and those with additional leads implanted, but later removed. Bilateral systems included patients with multiple leads implanted without concomitant lead removal and those with a *bilateral* CPT modifier associated with the IPG implant. Bilateral systems were classified as *non-staged* when both leads were implanted on the same day and *staged* when leads were implanted on different days during the acute period. The number of IPGs implanted within the acute period was recorded. Use of microelectrode recording (MER) was recorded based on the distinct CPT codes (Table 1).

Implanting center characteristics were derived from the CMS database. The implanting center was determined from the organization national provider identifier (NPI) associated with the index lead implant. Centers with at least one implant in both MD and TO subgroups were referred to as *Common Centers*. The number of *de novo* implants occurring at each center over the enrollment period was calculated, and centers within the top 20% were classified as *High-Volume Centers*. Of note, Common Center and High-Volume Center classifications are not mutually exclusive—a single center may fulfill criteria for both subgroups.

### Outcome Measures and Statistical Analysis

The primary outcome was DBS complications necessitating surgical revision or removal at any time during the study period. Revisions and removals were considered collectively as *reoperations*. Of note, CPT codes are different for end of service IPG replacements and were not considered a reoperation. In addition, the lead revision code is specific to the intracranial portion of the implant and is not intended for revision of the extensions or adaptors—such procedures have no specific code. Secondary outcome measures involved analysis of lead and IPG reoperations separately.

Diagnosis codes associated with each reoperation were used to determine the clinical reason for reoperation (Table 1). Based on available diagnoses, the reasons for reoperation were classified either as *infection, hardware malfunction*, or *other/unidentified*. Diagnoses within the other/unidentified category consisted primarily of neurological complications and unclear diagnoses that could not be reliably classified.

Kaplan–Meier analysis was performed to compare event-free survival rates between MD and TO subgroups. Cumulative hazard was determined using a Nelson–Aalen estimator. Unadjusted survival and event rates for lead-only, IPG-only, and combined reoperations were found for the acute period, 1-year post-index, and 2-year post-index. Survival and cumulative event models were computed using a Cox proportional hazard model with and without Andersen–Gill modification; and with adjustment for implant indication, age, sex, comorbidities, implantation year, unilateral/bilateral system, staged lead implant, and number of IPGs implanted in the acute period. Hazard ratios (HRs) and 95% CI were reported for hazard differences between the two subgroups, with HR < 1.0 indicating a lower risk in the MD subgroup. Event-free survival and cumulative event subanalyses were performed based on the diagnoses associated with reoperations. Subgroup analyses using Kaplan–Meier, Anderson–Gill, univariate, and multivariate regression models were used to determine the influence of patient, implant, and center characteristics. With missing data, analysis only included patients with available data and the *n* involved in the subanalysis is reported.

All analyses were conducted on the CMS VRDC using the SAS Enterprise Guide Version 7.15 HF3 (SAS Institute Inc., Cary, NC, USA). The CMS data privacy policy requires suppression when actual number of patients or events is fewer than 11. In such instances, results are limited to values of “*n* < 11” or the equivalent computed percentage ceiling for the cohort of interest.

## Results

**Figure 1:**
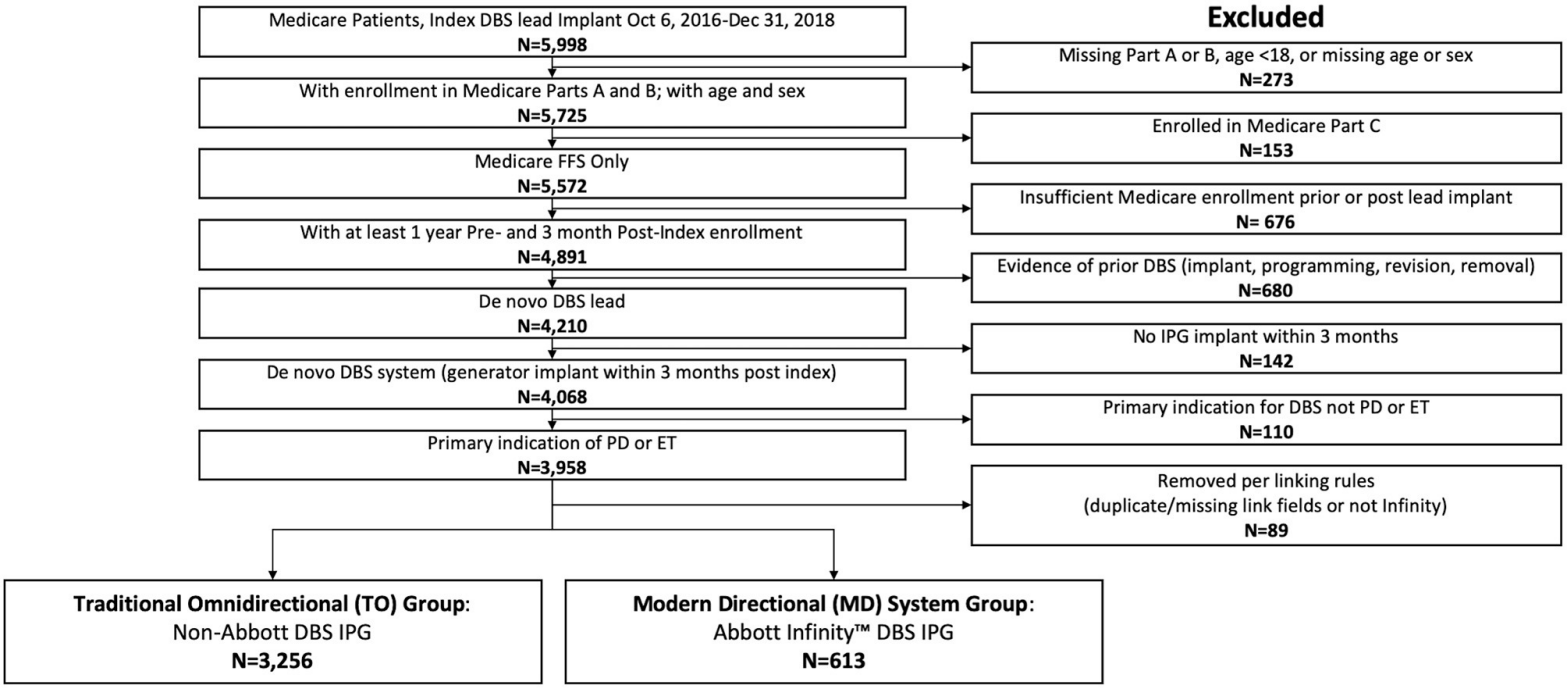
CONSORT flow diagram for this study. Of 5,998 patients implanted with a DBS system between October 6, 2016 and December 31, 2018, 3,869 patients were enrolled in Medicare Parts A and B, had sufficient follow-up, and were classified as a *de novo* implant for Parkinson's disease (PD) or essential tremor (ET) and were therefore eligible for analysis.

Of 5,998 patients who underwent DBS lead implantation between October 6, 2016 and December 31, 2018, 3,869 patients met inclusion and exclusion criteria (Figure 1): 3,256 (84.2%) in the TO subgroup and 613 (15.8%) in the MD subgroup. This distribution of *de novo* system implants is representative of practice patterns during the aforementioned timeframe.

There was no statistical difference in baseline demographics or in clinically relevant comorbidities between subgroups (Table 2). As such, matching of data between subgroups was unnecessary for statistical analysis.

**Table 2:** Patient demographics and selected comorbidities of clinical importance for the cohort and subgroups.

	**Total**	**Omnidirectional system group**	**Directional system group**	***P*-value**
	***n* = 3,869**	***n* = 3,256**	***n* = 613**	
**Age (years)**	70.9 ± 6.9	70.9 ± 6.9	70.9 ± 7.1	0.950
Age ≥65	3,355 (86.7%)	2,829 (86.9%)	526 (85.8%)	0.471
Age ≥75	1,027 (26.5%)	867 (26.6%)	160 (26.1%)	0.786
**Female sex**	1,476 (38.1%)	1,242 (38.1%)	234 (38.2%)	0.990
**Indication**				0.262
PD	2,513 (65.0%)	2,127 (65.3%)	386 (63.0%)	
ET	1,356 (35.0%)	1,129 (34.7%)	227 (37.0%)	
**Race**				0.181
Asian	–	35 (1.1%)	<11 (<1.8%)	
Black	51 (1.3%)	36 (1.1%)	15 (2.4%)	
Hispanic	–	46 (1.4%)	<11 (<1.8%)	
North American Native	–	<11 (<0.3%)	<11 (<1.8%)	
White	3,528 (91.2%)	2,977 (91.4%)	551 (89.9%)	
Other	58 (1.5%)	47 (1.4%)	11 (1.8%)	
Unknown	125 (3.2%)	106 (3.3%)	19 (3.1%)	
**Chronic conditions warehouse comorbidities**				
Atrial fibrillation	282 (7.3%)	238 (7.3%)	44 (7.2%)	0.908
Diabetes	998 (25.8%)	826 (25.4%)	172 (28.1%)	0.163
Heart failure	337 (8.7%)	285 (8.8%)	52 (8.5%)	0.828
Hypertension	2,324 (60.1%)	1,948 (59.8%)	376 (61.3%)	0.484
Stroke/transient Ischemic attack	208 (5.4%)	175 (5.4%)	33 (5.4%)	0.993
**Elixhauser comorbidities**				
Obesity	911 (23.5%)	767 (23.6%)	144 (23.5%)	0.972
Weight loss	381 (8.2%)	257 (7.9%)	61 (10.0%)	0.089

*Age is represented as a mean and SD. Distributions of other demographic information are reported as the raw number of patients and the corresponding percentage of patients. CMS data privacy policy requires suppression when actual number of patients or events is fewer than 11. In such instances, results are limited to values of “n <11” or the equivalent computed percentage ceiling for the cohort of interest. The p-value represents the significance of a t-test for continuous variables or the chi-square test for categorical variables between the subgroups*.

Implants occurred at 283 unique centers. Mean follow-up post-index was 626 ± 140 days, with patients in the TO subgroup having a significantly longer follow-up duration (*p* < 0.001). Full details regarding the implant characteristics are shown in Table 3.

**Table 3:** Implant characteristics for the traditional omnidirectional subgroup and the modern directional subgroup.

	**Traditional omnidirectional system group *n* = 3,256**	**Modern directional system group *n* = 613**	***P*-value**
Follow-up duration (days)	633 ± 138	585 ± 145	<0.001
Bilateral lead	2,201 (67.6%)	447 (72.9%)	0.009
Implanted at common centers	1,179 (36.2%)	530 (86.5%)	<0.001
Implanted at high volume centers (upper 20%, ≥21 implants)	1,740 (53.4%)	314 (51.2%)	0.590
Implanted with MER	2,329 (71.5%)	453 (73.9%)	0.473

*MER, microelectrode recording*.

*Follow-up is represented as a mean and SD. Distributions of other details are reported as the raw number of patients and the corresponding percentage of patients. The p-value represents the significance of a t-test for continuous variables or the chi-square test for categorical variables between the subgroups. Of note, Common Center and High-Volume Center classifications are not mutually exclusive; as such, the sum of these subgroups is not expected to total to 100%*.

### Primary Outcomes

Of the 3,869 study patients, 379 (9.8%) required reoperation within 2 years. Kaplan–Meier estimate of reoperations was 10.7% for the TO subgroup and 7.8% for the MD subgroup (Table 4).

**Table 4:** Rate of patients experiencing complications at selected time points, based on unadjusted Kaplan–Meier analysis (^*^indicates suppression of cells with fewer than 11 counts, per Centers for Medicare and Medicaid Services policy).

	**Omnidirectional system group** ***n*** **=** **3,256**			**Directional system group** ***n*** **=** **613**			***p*-value**
	**3 months**	**1 year**	**2 years**	**3 months**	**1 year**	**2 years**	
Lead and IPG	5.44%	8.68%	10.73%	2.28%	6.28%	7.76%	0.033
Lead only	4.79%	7.51%	9.11%	<1.8%*	4.97%	6.57%	0.031
IPG only	1.69%	3.82%	5.18%	<1.8%*	3.15%	3.96%	0.294

*IPG, implantable pulse generator*.

*The p-value represents the significance of a t-test between the subgroups*.

**Figure 2:**
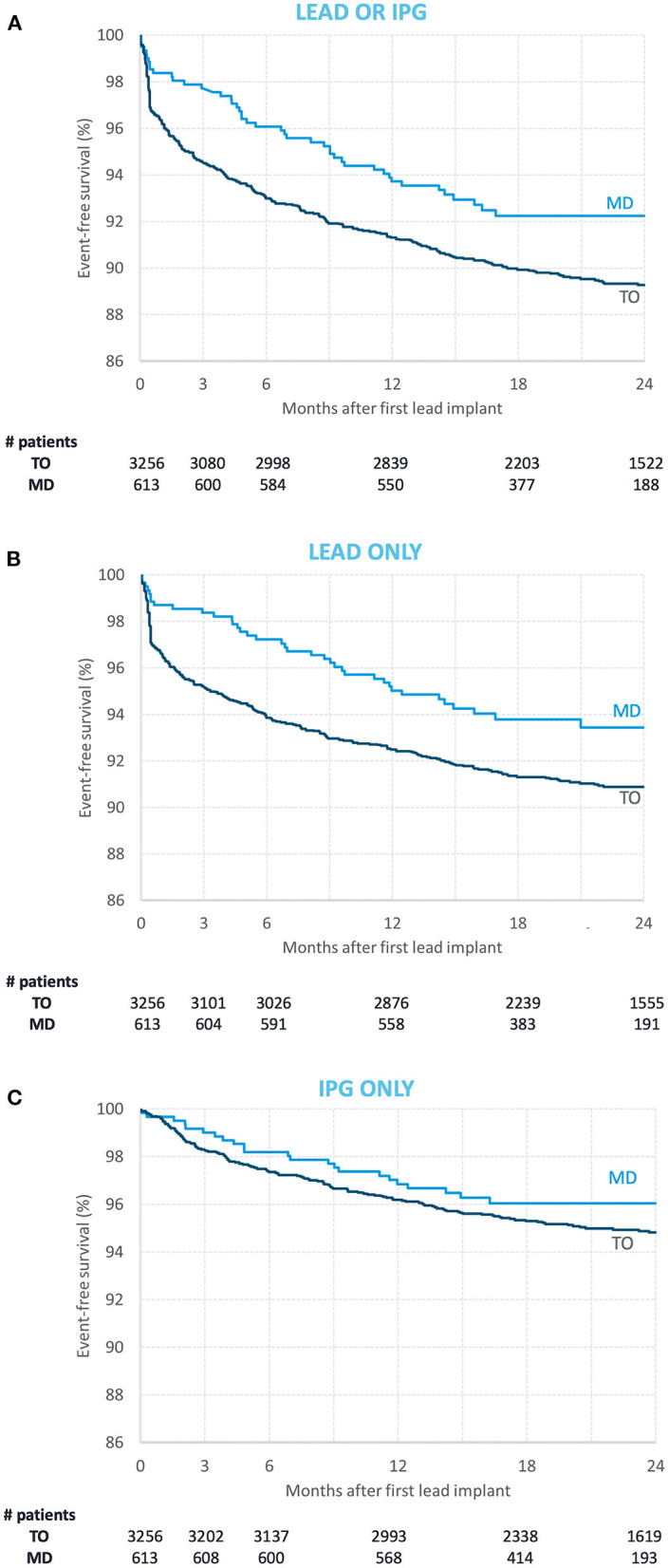
Event-free survival curves for the modern directional (MD) subgroup (light blue) and the traditional omnidirectional (TO) subgroup [dark blue] with regards to **(A)** lead and implantable pulse generator (IPG) reoperations combined, **(B)** lead only reoperations, and **(C)** IPG only reoperations. The number of subjects in each subgroup at index implantation and at 3-, 6-, 12-, 18-, and 24-month follow-up are reported below each graph.

The event-free survival curves (Figure 2) show that a significant portion of reoperations occurred within the acute period (5.4% of patients in the TO subgroup vs. 2.3% of patients in the MD subgroup).

After adjustment for potential confounders, patients in the MD subgroup were 36% less likely to require reoperation (HR = 0.64, 95%CI [0.47, 0.88], *p* = 0.007). This difference was more evident for lead reoperations (HR = 0.60, 95%CI [0.42, 0.85], *p* = 0.005) than for IPG reoperations (HR = 0.81, 95%CI [0.52, 1.26], *p* = 0.36).

### Reasons for Reoperation

For the 412 lead reoperations, 123 (30%) were associated with infection, 100 (24.2%) with hardware malfunction, and 189 (45.9%) were associated with other/unclassified reasons. Of the 216 IPG reoperations, 100 (46.3%) were associated with infection, 62 (28.7%) with hardware malfunction, and 54 (25.0%) were associated with other/unclassified reasons. Lead reoperation for other/unclassified reasons was significantly lower in patients in the MD subgroup (HR = 0.44, 95%CI [0.24, 0.81], *p* = 0.008), but other classifications were not significantly different between the two subgroups. Interestingly, 112 (63.6%) of the 176 lead reoperations performed in the acute period were associated with other/unclassified reasons, 41 (63.1%) of the 65 IPG reoperations in the acute period were associated with infections. In the chronic phase, the associated diagnoses were balanced across all the three categories.

### Characteristics of Involved Centers

Among the 283 centers, 272 (96.1%) implanted TO DBS systems and 102 (36.0%) implanted MD DBS systems. There were 91 (32.2%) Common Centers implanting both system types, which accounted for 1,709 (44.2%) of the entire study cohort. The implanting center could not be determined in 339 (8.8%) patients because there was no organization NPI associated with the index implant. Implant procedure volume for individual centers ranged from 1 to 122 *de novo* implants over the 27-month enrollment period. About 57 centers implanting at least 21 patients with Medicare coverage were classified as High-Volume Centers, which performed 36.0 ± 16.8 *de novo* implants over the entire study period. The remaining centers performed 6.5 ± 5.4 implants.

Of 412 lead reoperations, there were 137 (33.3%) procedures for which it could not be determined whether the reoperation occurred at the same center as the index lead implant. Of the remaining 275 procedures, 24 (8.7%) reoperations occurred at a different center than the index implant. Likewise, of the 216 IPG reoperations, there were 88 (40.7%) procedures for which it could not be determined whether the reoperation occurred at the same center as the index lead implant. Of the remaining 128 procedures, 17 (13.3%) reoperations occurred at a different center.

### Influence of Covariates

Age, sex, comorbidities, implant characteristics, and center characteristics did not have statistically significant interactions with the primary outcome of reoperations. Only implant indication (PD vs. ET) demonstrated a significant interaction: patients implanted for PD in the MD subgroup had fewer reoperations (HR = 0.47, 95%CI [0.30, 0.74], *p* = 0.001), whereas for patients implanted for ET, the reoperation risk was comparable (HR = 0.96, 95%CI [0.61, 1.51], *p* = 0.861).

**Figure 3:**
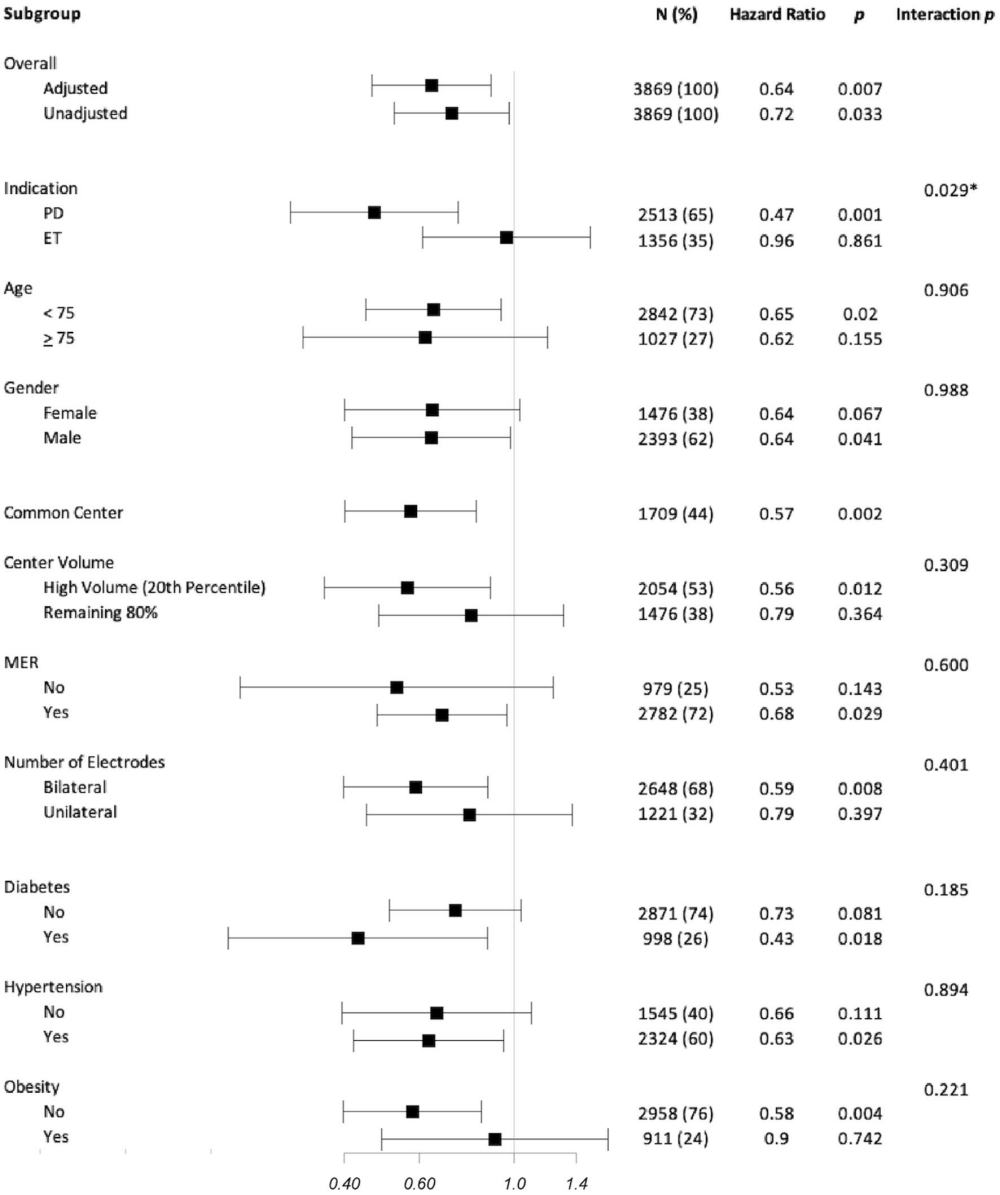
Subgroup analysis of hazard ratio (HR) for event-free survival from lead or implantable pulse generator reoperations. HRs are represented as squares with 95% CI bars. HRs <1 represents a reduced risk associated with modern directional systems. Columns show the number of subjects included in the subgroup analysis is reported for each analysis; the equivalent percentage of the entire cohort that this subgroup represents is reported in parentheses. Within the subgroup analysis, only the primary diagnosis associated with the DBS implant demonstrated a statistically significant difference (indicated by an *) with those implanted for Parkinson's disease experiencing a notable HR reduction compared to those implanted for essential tremor. There was no significant difference when considering age, gender, center, center experience, use of MERs, number of electrodes implanted, or comorbidities of diabetes, hypertension, or obesity.

Among Common Centers, the reoperation risk remained lower for patients in the MD subgroup (HR = 0.57, 95%CI [0.40, 0.82], *p* = 0.002). This relative risk reduction was also present in High-Volume Centers (HR = 0.56, 95%CI [0.36, 0.88], *p* = 0.012), but was not statistically significant for the remaining centers (HR = 0.79, 95%CI [0.48, 1.31], *p* = 0.364). Figure 3 shows the HR of event-free survival across subgroups.

## Discussion

### Modern Directional DBS Systems Are Associated With Fewer Reoperations

Using US insurance claims linked with patient device tracking data, we found that DBS reoperations occurred 36% less frequently within 2 years of follow-up with MD systems than with TO systems. This reduction in cumulative hazard over the entire study period is clinically relevant, as it represents the overall risk to patients over long-term follow-up.

Separate analysis of reoperation rates for leads and IPGs demonstrates a similar relationship for the former, but a weaker association for the latter. As such, it appears that the primary factor in risk reduction seen with MD systems is the difference in lead-related complications. The unique features of stimulation field shaping and more contemporary design and construction with MD system leads may therefore play a significant role in reoperation risk.

### Suboptimal Electrode Location may Play a Significant Role in Reoperations

Neither infection nor hardware malfunction rates were significantly different between the two subgroups; however, for patients who underwent reoperation for other/unclassified reason, there was a 56% risk reduction in the MD subgroup. This classification was the most common reason for lead reoperations in the cohort. As suggested by Rolston et al. (10), this other/unclassified reason for reoperation is likely attributable to mispositioned electrodes or lack of therapeutic effect, as there is no other clear clinical indication for reoperation (10). This interpretation supports the hypothesis that stimulation field shaping, which has been shown to expand the therapeutic window for stimulation (4), significantly reduces reoperation rates.

Interestingly, we see that the primary difference between reoperation rates occurred in the acute period. Such timing of reoperations has been described in prior analysis of healthcare claims data (11). With the difference between subgroups occurring early, it is unlikely that our results are meaningfully affected by a mean difference in follow-up of 48 days in the setting of a mean total follow-up of 626 days. About 42.7% of all lead reoperations occurred within this acute period, for which the majority (63.6%) may be attributable to suboptimal electrode placement. Such a meaningful difference in early reoperations raises the question of whether clinician thresholds for reoperation differ between the two systems. While nuances of clinical decision-making cannot be captured in our analysis, the long-term follow-up data provides some insight. After the acute period, the infection and hardware malfunction were more common for lead reoperations and MD systems to maintain their relative risk reduction over time. These findings suggest MD systems have lasting safety benefits and do not simply delay reoperations beyond the acute period.

### Risk Reduction Persists Across Implant Methodologies and Center Experience

We wanted to account for the possible influence of implantation technique on reoperations. We therefore performed a subanalysis with Common Centers to exclude patients who might have undergone an implant methodology not represented in both subgroups. These centers demonstrated a similar pattern of relative risk reduction. Furthermore, specific procedural variables, including use of MER and number of electrodes implanted, did not significantly influence the risk reduction. Although we were unable to control for every variable in surgical technique, this approach broadly mitigates practice differences across centers.

Given that MD systems were introduced at the beginning of the study period, we were concerned about the possibility that only more experienced centers would utilize newer technology and possibly bias the results. We, therefore, used volume of implantation as a surrogate for experience and performed a subanalysis based on center volume. Although the threshold for classification as a High-Volume Center was 21 *de novo* implantations, it is important to recognize that our cohort consists only of those under Medicare coverage. As such, when considering all insurance carriers, High-Volume Centers are estimated to have performed at least twice as many implants. Nevertheless, center volume had no significant impact on the overall relative risk reduction. It is, however, notable that there was a trend toward greater risk reduction in High-Volume Centers. This finding is particularly interesting when considering that High-Volume Centers are also more likely to treat more complex patients. It is possible that greater attention paid to surgical procedures associated with a novel device could contribute to a lower complication rate.

### Patients With Parkinson's Disease Experience Greater Risk Reduction

Patients undergoing DBS for PD demonstrated greater relative risk reduction with MD systems than those implanted for ET. While age and comorbidities may be different between these cohorts, these factors did not impact reoperation rates in our analysis. A potential reason for this difference is that the thalamic target used for ET is more forgiving to off-target effects than the commonly used subthalamic nucleus target used for PD. Without DBS targets in the CMS database, we were unable to investigate this possibility. Nevertheless, this PD-specific risk reduction makes it less likely that differential treatment of a novel system explains the risk reduction associated with MD systems, as practice differences would similarly affect those with ET.

### Not all Reoperations Occur at the Original Implanting Center

The ability to longitudinally track patients independent of institutional data revealed that patients may undergo reoperation at a different center. This phenomenon is important to consider, as it may lead to an underestimation of complication rates if patient are lost to follow-up in single center analyses.

### Comparison With Published DBS Complication Rates

A recent systematic review noted that 3.8% of patients underwent hardware removal and 4.5% underwent hardware revision (3). The authors also presented evidence for a publication bias toward lower complication rates. Meanwhile, healthcare claims data offer large sample sizes free of selection bias that are representative of different practice patterns (12). Investigation of US healthcare claims data has revealed slightly higher complication rates with lead and IPG revision rates within 90 days of implantation of 5.3 and 3.2%, respectively (11). Such databases also provide greater longitudinal tracking, which has revealed that complication rates may increase with time. Rolston et al. (10) reported reoperation procedures represented 15.2% of all DBS surgeries in a 10-year period—a statistic likely including complications in patients implanted before the study period (10). It is therefore reasonable that while the reoperation rate in the present study was 5.4% at 3 months, it increased to 9.8% by 24 months.

### Limitations

While there is a wealth of data in the CMS database, we are still limited to the information that has been captured for billing purposes (12). As such, we could not identify complications that did not require reoperation. Similar to other retrospective studies, misclassifications or misdiagnoses may occur when the most suitable procedure or diagnosis code was not submitted. Since revision of extensions or adapters to not have a specific code, it is possible that such procedures were coded as either a lead revision or an IPG revision. Without the ability to prospectively randomize patients, we were unable to control all confounding variables. We also lacked access to richer clinical data, which may provide a more complete picture of the clinical scenario. As such, adjudication of specific reasons for reoperation was not always possible. While infection and hardware malfunction could be identified, we were unable to classify a significant proportion of events. Since there is no dedicated code for lack of therapeutic efficacy, it is reasonable to interpret other/unclassified diagnoses as instances where DBS resulted in side effects or lack of efficacy, particularly for lead reoperations. Unfortunately, we cannot definitely consider this etiology as the sole reason for such reoperations.

In terms of generalizability, our study only included patients with Medicare coverage, which is only available to patients over the age of 65. We therefore cannot extend conclusions to younger patients or those without Medicare coverage.

While providing considerable insight into DBS reoperations, our study also generates several questions about the reasons underlying the observed findings.

## Conclusions

This is the first multicenter real-world report of device-related complications with modern DBS systems. MD systems were significantly less likely to require reoperation, even when accounting for practice differences and implanting center experience. This relative risk reduction was seen primarily in patients with PD and was largely attributable to the reduction in lead reoperations. This differential risk profile should be carefully considered during device selection for patients undergoing DBS for PD or ET. Further studies are needed to investigate the underlying causes of DBS reoperations in order to better understand the underpinnings of the risk reduction associated with MD systems.

## Data Availability Statement

Centers for Medicare and Medicaid Services data can be requested under an approved research protocol via ResDAC (www.resdac.org). Restrictions apply to the availability of data generated and analyzed during this study to preserve patient confidentiality and because data are used under license. Requests to access these datasets should be directed to ResDAC, resdac@umn.edu.

## Ethics Statement

The studies involving human participants were reviewed and approved by Western IRB. Written informed consent for participation was not required for this study in accordance with the national legislation and the institutional requirements.

## Author Contributions

CW, RA, BC, and PL contributed to the concept and design of the study. CW, RA, and BC helped in acquisition and verification of data. CW, SN, RA, MP-N, WH, AS, AC, BC, and PL interpreted the data. CW drafted the manuscript and has full access to all the data in the study and is responsible for the integrity of the data and the accuracy of the data analysis. SN, RA, MP-N, WH, AS, AC, BC, and PL critically revised the manuscript for important intellectual content. AC and RA performed statistical analysis. BC and PL supervised the study. All authors contributed to the article and approved the submitted version.

## Funding

Abbott provided funding for this study.

## Conflict of Interest

CW, SN, MP-N, WH, and PL are paid consultants for Abbott Laboratories. RA, AC, and BC are employees of Abbott Laboratories. The remaining author declares that the research was conducted in the absence of any commercial or financial relationships that could be construed as a potential conflict of interest. The authors declare that this study received funding from Abbott Laboratories. The funder was not involved in the study design, collection, analysis, interpretation of data, the writing of this article or the decision to submit it for publication.

## Publisher's Note

All claims expressed in this article are solely those of the authors and do not necessarily represent those of their affiliated organizations, or those of the publisher, the editors and the reviewers. Any product that may be evaluated in this article, or claim that may be made by its manufacturer, is not guaranteed or endorsed by the publisher.

## Abbreviations

CCW: Chronic Conditions Warehouse
CMS: Centers for Medicare and Medicaid Services
CPT: Current Procedural Terminology
DBS: deep brain stimulation
ET: essential tremor
HR: hazard ratio
ICD: International Classification of Diseases
IPG: implantable pulse generator
MBSF: Master Beneficiary Summary File
MER: microelectrode recording
MD: modern directional
NPI: national provider identifier
PD: Parkinson's disease
PDT: Patient Device Tracking
TO: traditional omnidirectional
VRDC: Virtual Research Data Center.

## References

[1] Bratsos SP Karponis D Saleh SN. Efficacy and safety of deep brain stimulation in the treatment of Parkinson's disease: a systematic review and meta-analysis of randomized controlled trials. Cureus. (2018) 10:e3474. 10.7759/cureus.3474 30648026 PMC6318091

[2] Lange M Mauerer J Schlaier J. Underutilization of deep brain stimulation for Parkinson's disease? A survey on possible clinical reasons. Acta Neurochir. (2017) 159:771–8. 10.1007/s00701-017-3122-3 28258308

[3] Engel K Huckhagel T Gulberti A Pötter-Nerger M Vettorazzi E Hidding U. Towards unambiguous reporting of complications related to deep brain stimulation surgery: a retrospective single-center analysis and systematic review of the literature. PLoS ONE. (2018) 13:e0198529. 10.1371/journal.pone.0198529 30071021 PMC6071984

[4] Schnitzler AS Mir PM Brodsky MA Verhagen L Groppa S Alvarez R. Directional versus omnidirectional deep brain stimulation for Parkinson's disease: results of a prospective, blinded, multi-center, single-arm crossover study. Mov Disord. (2019) 34 (Suppl. 2).

[5] Steigerwald F Matthies C Volkmann J. Directional deep brain stimulation. Neurotherapeutics. (2019) 16:100–4. 10.1007/s13311-018-0667-7 30232718 PMC6361058

[6] Centers for Medicare Medicaid Services. ResDAC. Available online at: https://www.resdac.org/ (accessed August 10, 2020).

[7] Hammill BG Hernandez AF Peterson ED Fonarow GC Schulman KA Curtis LH. Linking inpatient clinical registry data to Medicare claims data using indirect identifiers. Am Heart J. (2009) 157:995–1000. 10.1016/j.ahj.2009.04.002 19464409 PMC2732025

[8] Elixhauser A Steiner C Harris DR Coffey RM. Comorbidity measures for use with administrative data. Med Care. (1998) 36:8–27. 10.1097/00005650-199801000-00004 9431328

[9] Centers for Medicare and Medicaid Services. CCW Medicare Administrative Data User Guide (Version 3.6). Chronic Condition Data Warehouse. Available online at: https://www2.ccwdata.org/documents/10280/19002246/ccw-medicare-data-user-guide.pdf (accessed August 12, 2020).

[10] Rolston JD Englot DJ Starr PA Larson PS. An unexpectedly high rate of revisions and removals in deep brain stimulation surgery: analysis of multiple databases. Park Relat Disord. (2016) 33:72–7. 10.1016/j.parkreldis.2016.09.014 27645504 PMC5240785

[11] Petraglia FW Farber SH Han JL Verla T Gallis J Lokhnygina Y. Comparison of bilateral vs. staged unilateral Deep Brain Stimulation (DBS) in Parkinson's disease in patients under 70 years of age. Neuromodulation Technol Neural Interface. (2016) 19:31–7. 10.1111/ner.12351 26568568 PMC4724316

[12] Stein JD Lum F Lee PP Rich WL Coleman AL. Use of health care claims data to study patients with ophthalmologic conditions. Ophthalmology. (2014) 121:1134–41. 10.1016/j.ophtha.2013.11.038 24433971 PMC4012019

